# Pharmacological management of tourette's syndrome comorbid with obsessive-compulsive disorder in adult patients

**DOI:** 10.1192/bjo.2021.788

**Published:** 2021-06-18

**Authors:** Himanshu Tyagi, Olabisi Ogunbiyi

**Affiliations:** National Hospital for Neurology and Neurosurgery

## Abstract

**Aims:**

Tourette's Syndrome (TS) is a neurodevelopmental disorder, which often presents in childhood and is hallmarked by motor and vocal tics. Obsessive-Compulsive Disorder (OCD) is a chronic neuropsychiatric condition characterised by intrusive thoughts and time-consuming repetitive behaviours. Research suggests that 15-20% of adult patients with TS will also meet the diagnostic criteria for OCD. Both illnesses appear to have neurobiological similarities but a differing course and clinical response to pharmacological treatments.

Despite this, research into optimal management of adults with co-occurring TS or other tic disorders and OCD remains sparse. Comorbidities, are known to be poor predictor of response to selective serotonin reuptake inhibitors (SSRI) monotherapy in OCD and are often associated with treatment-refractory OCD. Similarly SSRI monotherapy in patients with OCD and comorbid TS can sometimes worsen motor tics (1 in 2000) and fail to improve OCD symptoms. In this review, we aim to evaluate evidence on the management of patients with co-occurring TS and OCD and address an important knowledge gap in clinical practice.

**Method:**

This review was conducted in accordance with PRISMA Guidelines. We performed a search using PubMed, Cochrane Library and PsychINFO using the following Boolean Input “Tourettic-OCD” OR “tic-related OCD” OR ((OCD OR “obsessive-compulsive” OR “obsessive compulsive”) AND (Tourette OR “Tourette's” OR Tourettes OR tic))”. The search was conducted until January 2020. We then screened the articles of systematic reviews to extract additional studies from their reference lists.

**Result:**

1888 studies were identified, of which 15 clinical trials were included in our systematic review. The presence of tics in patients with OCD are a major predictor for treatment-refractory OCD and a lack of improvement following monotherapy with SSRIs. Dual therapy with an SSRI and antipsychotics (particularly risperidone) are associated with improved outcomes in OCD patients with tics and TS patients with obsessive-compulsive symptoms. However, conjoint therapy with neuroleptics and SSRIs was only investigated when OCD burden was unsatisfactory following SSRI monotherapy.

**Conclusion:**

There are clinical implications when a patient with OCD also has a chronic tic disorder. The findings indicate the need for further research, particularly in the form of a larger cohort in randomised controlled trials, to determine when it is best to initiate patients with OCD and comorbid tic disorders on a dual antipsychotic-SSRI management strategy. Further evidence should also be done to determine other characteristics that predict an improvement to conjoint SSRI/neuroleptic therapy for effective symptom reduction.

